# Supplementary material for: Impact of lockdown on COVID-19 epidemic in Île-de-France and possible exit strategies
Source: BMC Med. 2020 Jul 30;18:240. doi: 10.1186/s12916-020-01698-4 (PMC7391016; doi:10.1186/s12916-020-01698-4)
Supplement: Supplementary file 1 — Additional file 1. Supplementary material composed of supplementary results, Tables S1 and S2, and Figures S1-S10. [file 12916_2020_1698_MOESM1_ESM.pdf]

ADDITIONAL FILE 1

Impact of lockdown on COVID-19 epidemic in Île-de-France and possible exit strategies

Laura Di Domenico[1], Giulia Pullano[1,2], Chiara E. Sabbatini[1], Pierre-Yves Boëlle[1], Vittoria Colizza[1]

1 INSERM, Sorbonne Université, Pierre Louis Institute of Epidemiology and Public Health, Paris, France
2 Sociology and Economics of Networks and Services lab at Orange Experience Design Lab (SENSE/XDLab) Chatillion, Paris, France

1. MODEL PARAMETERS 2
1.1. Compartmental model 2
1.2. Generation time distribution 3
1.3. Estimation of within-hospital parameters 4
1.4. Mixing matrices 5
2. MODEL CALIBRATION 6
3. ADDITIONAL RESULTS 7
3.1. Consultation rate throughout lockdown 7
3.2. Lockdown lifted at the beginning of May 8
3.3. Physical contacts avoided throughout the exit strategies 9
4. SENSITIVITY ANALYSIS 9
4.1. Probability of being asymptomatic 50% 9
4.2. Relative susceptibility of children 13
4.3. Relative infectivity of younger children 14

1. MODEL PARAMETERS

1.1. Compartmental model

Table S1. Parameters, values, and sources used to define the compartmental model

Variable	Description	Value	Source
θ^{-1}	Incubation period	5.2d	[30]
μ_p^{-1}	Duration of prodromal phase	1.5d, computed as the fraction of pre-symptomatic transmission events out of pre-symptomatic plus symptomatic transmission events.	[31]
ϵ^{-1}	Latency period	$\theta^{-1} - \mu_p^{-1}$	-
p_a	Probability of being asymptomatic	0.2, 0.5	[21]
p_{ps}	If symptomatic, probability of being paucisymptomatic	1 for children<br>0.2 for adults, seniors	[18]
p_{ms}	If symptomatic, probability of developing mild symptoms	0 for children<br>0.7 for adults<br>0.6 for seniors	[18]
p_{ss}	If symptomatic, probability of developing severe symptoms	0 for children<br>0.1 for adults<br>0.2 for seniors	[18,19,33]
g	Generation time	6.6d	[34]
μ^{-1}	Infectious period for I_a, I_{ps}, I_{ms} , I_{ss}	2.3d, chosen accordingly to generation time distribution (see following subsection)	-
r_β	Relative infectiousness of I_p, I_a, I_{ps}	0.55	[8]
p_{ICU}	If severe symptoms, probability of going in ICU	0 for children<br>0.24 for adults<br>0.24 for seniors	[28]
$\lambda_{H,R}$	If hospitalized, daily rate entering in R	0 for children<br>0.083 for adults<br>0.033 for seniors	[28]
$\lambda_{H,D}$	If hospitalized, daily rate entering in D	0 for children<br>0.0031 for adults<br>0.0155 for seniors	[28]
$p_{ICU,R}$	Probability of recovery from ICU	0.76 for adults<br>0.54 for seniors	[28]
λ_{ICU}^{-1}	Time spent in ICU	21.1 days for adults<br>20.7 days for seniors	[28]

1.2. Generation time distribution

The generation time distribution in a compartmental epidemic model can be computed thanks to the theory developed by Svensson[32]. Let X and Y be the random variables describing the latency period and the infectious period, respectively. Then the distribution of the generation time is the result of the convolution $g * h_s$, with g being the probability density function of X and

$$h_s(t) = \frac{1 - H(t)}{E(Y)}$$

where H is the cumulative distribution function of Y, and $E(Y)$ is the mean.
In the compartmental model under consideration (Figure 2), we have that X is exponentially distributed with rate ϵ, and Y is the sum of two exponentially distributed random variables (prodromic phase and infectious period, with rate μ_p and μ respectively). Computations show that the corresponding generation time distribution is

$$f(t) = \frac{\epsilon\, \mu_p\, \mu}{(\mu_p + \mu)(\mu - \mu_p)} \left[\frac{\mu}{(\epsilon - \mu_p)} \left(e^{-\mu_p t} - e^{-\epsilon\, t}\right) - \frac{\mu_p}{(\epsilon - \mu)} \left(e^{-\mu\, t} - e^{-\epsilon\, t}\right)\right]$$

Given the values of ϵ and μ_p informed from the literature (Table S1), we choose μ so that the mean of the generation time equals to 6.6 days. The shape of the distribution is displayed in Figure S1 and it closely resembles a gamma distribution with mean 6.6 and shape parameter 1.87, estimated in Ref.[34]

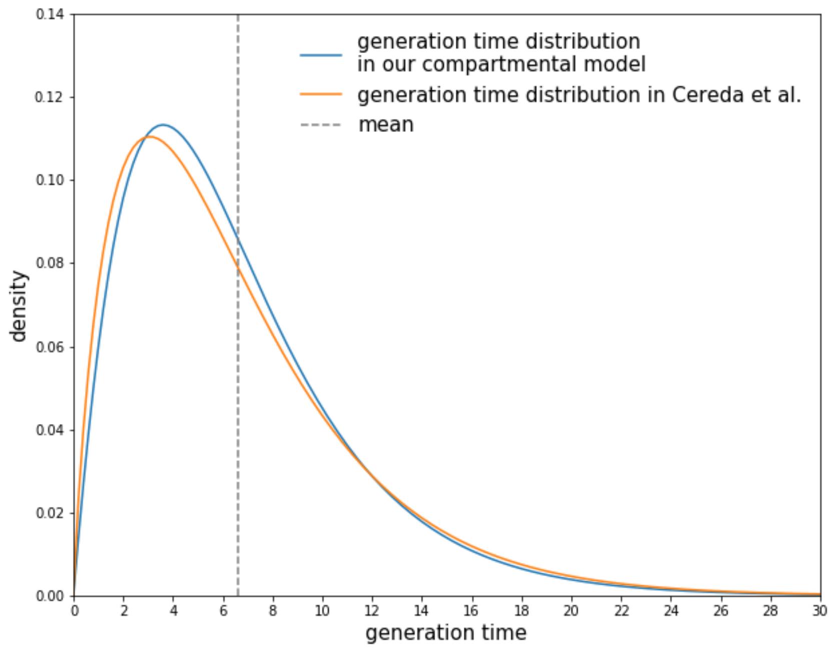

Figure S1. Distribution of the generation time. The generation time distribution corresponding to our compartmental model (blue) in comparison with the distribution estimated in Ref.[34] (orange).

1.3. Estimation of within-hospital parameters

We fit data on patient trajectories recorded in Île-de-France hospitals after admission up to April 5, 2020. Data consisted of age, sex, date of hospital admission and subsequent dates of discharge or death, and, when relevant, dates of entering/leaving the ICU. We fit mixture and competing risks models to time to event data, taking into account censoring due to patients being still in the hospital at the time of analysis. We used exponential distributions for time to event data to match the hypotheses of the compartmental epidemic model.

First, we model time from admission to entering the ICU or being discharged/dead for those who do not go to the ICU. Write T for the time to the first of the 3 following events: entering the ICU, being discharged alive or dying in the hospital. T is modelled as a mixture of 2 exponential distributions: T ~ $\pi_{ICU} Exp(\lambda_{ICU}) + (1-\pi_{ICU}) Exp(\lambda_H)$, where π_{ICU} is the probability to go to the ICU, and λ_{ICU}, λ_H are the rates of the exponential distributions. The second exponential describes time spent in the hospital by those who don't go the ICU subject to competition of 2 outcomes, discharge or death. Therefore, $\lambda_H = \lambda_{DIS} + \lambda_{DTH}$ where λ_{DIS} is the rate of discharge and λ_{DTH} the rate of death. The average time spent in the hospital is $1/\lambda_H$, and the probability of being discharged alive is $\lambda_{DIS}/(\lambda_{DIS} + \lambda_{DTH})$. Therefore, the likelihood of a patient trajectory observed up to time t with final status s (comprising still hospitalized - HOS, admitted to ICU - ICU, discharged alive - DIS, dead - DTH) is given by :

$$L(\pi_{ICU}, \lambda_{ICU}, \lambda_{DIS}, \lambda_{DTH}) = (\pi_{ICU} \lambda_{ICU} \exp(-\lambda_{ICU}\, t))^{s=ICU}$$

$$\left((1-\pi_{ICU})\,(\lambda_{DIS}/(\lambda_{DIS}+\lambda_{DTH}))^{s=DIS}\,(\lambda_{DTH}/(\lambda_{DIS}+\lambda_{DTH}))^{s=DTH} \exp(-(\lambda_{DIS}+\lambda_{DTH})t)\right)^{1-s=ICU}$$

$$(1-\pi_{ICU} \exp(-\lambda_{ICU}\, t) - (1-\pi_{ICU}) \exp\,(-(\lambda_{DIS}+\lambda_{DTH})\, t))^{s=HOS}$$

The first line is for patients going to the ICU, the second line for those being discharged alive or dead and the third line for patients who were censored because they were still in the hospital.

Likewise, we fit time to discharge or death after admission to the ICU using a competing risk approach with exponential parameters μ for being discharged alive or dead; the likelihood is therefore:

$$L(\mu_{DIS}, \mu_{DTH}) =$$

$$((\mu_{DIS}/(\mu_{DIS}+\mu_{DTH}))^{s=DIS}\,(\mu_{DTH}/(\mu_{DIS}+\mu_{DTH}))^{s=DTH} \exp(-(\mu_{DIS}+\mu_{DTH})t))^{1-s=ICU}$$

$$(1-\exp\,(-(\mu_{DIS}+\mu_{DTH})\, t))^{s=HOS}$$

As the data is rounded to the nearest day, we discretized the exponential distributions in the likelihood. All models were fitted at maximum likelihood using the software R.

Estimates up to April 5, 2020 were also compared to values estimated in the period April 5-26 to assess possible changes in the management of COVID-19 patients at the hospital. We report the values in Table S2. These estimates are discussed in the main paper, but are not included in the analysis, as they became available at a later time.

Table S2. Estimates of within-hospital parameters in two different periods of time.

	March 1 – April 5, 2020	April 5 – 26, 2020
p_{ICU}	0.24 for adults, seniors	0.16 for adults, seniors
$\lambda_{H,R}$	0.0832 for adults<br>0.0328 for seniors	0.0834 for adults<br>0.0330 for seniors
$\lambda_{H,D}$	0.0031 for adults<br>0.0155 for seniors	0.0023 for adults<br>0.0115 for seniors
$p_{ICU,R}$	0.76 for adults<br>0.54 for seniors	0.84 for adults<br>0.64 for seniors
λ_{ICU}^{-1}	21.1 days for adults<br>20.7 days for seniors	15.0 days for adults<br>16.6 days for seniors

1.4. Mixing matrices

Here we report the matrices computed for all interventions tested, compared to the baseline scenario.

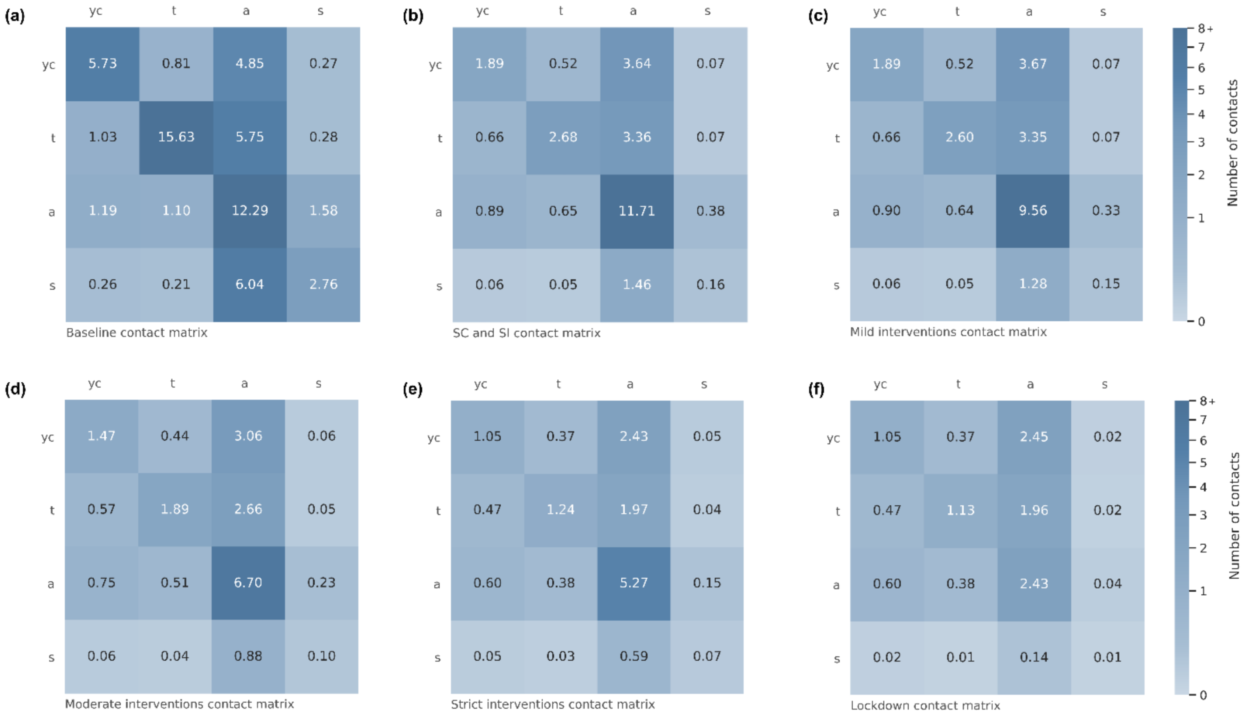

Figure S2. Mixing matrices for the baseline and all social distancing interventions tested. (a) Baseline contact matrix (b) School closure and senior isolation contact matrix (c) Mild interventions contact matrix (d) Moderate interventions contact matrix (e) Strict interventions contact matrix (f) Lockdown contact matrix.

2. MODEL CALIBRATION

The model was calibrated to hospital admission data through a maximum likelihood approach. The likelihood function is of the form

$$L(Data|\Theta) = \prod_{t=t_1}^{t_n} Poiss\left(H_{obs}(t)\middle|H_{pred}(t)\right)$$

where Θ indicates the set of parameters to be estimated, $H_{obs}(t)$ is the observed number of hospital admissions on day t, $H_{pred}(t)$ is the number of hospital admissions predicted by the model on day t, $Poiss\left(\cdot\middle|H_{pred}(t)\right)$ is the probability mass function of a Poisson distribution with mean $H_{pred}(t)$, and $[t_1, t_n]$ is the time window considered for the fit. We fit the transmission rate per contact before lockdown and the starting date of the simulation, considering a time window ranging from March 1 to March 23, 2020. Hospital admissions in the interval March 17-23 were included as still not affected by lockdown, due to delay between date of infection and date of hospitalization (~1 week). The resulting estimate of the transmission rate is 0.0791, with 95% CI [0.0769, 0.0806], corresponding to $R_0 = 3.18$ [3.09, 3.24].

Our model predictions were also compared to the ones obtained by fitting the model to hospital admission data during lockdown. We chose the period April 13-May 10 to avoid the initial fluctuations due to the implementation of lockdown. By doing that, we obtain an estimated transmission rate at 0.0833, corresponding to a 5.3% increase with respect to the transmission rate prior to lockdown.

The number of hospital admissions, ICU admissions and ICU occupancy over time for Ile-de-France (Figure 4a, Figure 5) are available in an open access repository (https://docs.google.com/spreadsheets/d/17Q5BlJw2N6b5uf8T2E3leTul91tAyqOvKhymvPDyhHk/edit?usp=sharing). They are part of the SIVIC database maintained by the Agence du Numérique en Santé and Santé Publique. The same online repository also contains the incidence of clinical cases estimated from sentinel and virological surveillance by Réseau Sentinelles (Figure 4b).

3. ADDITIONAL RESULTS

3.1. Consultation rate throughout lockdown

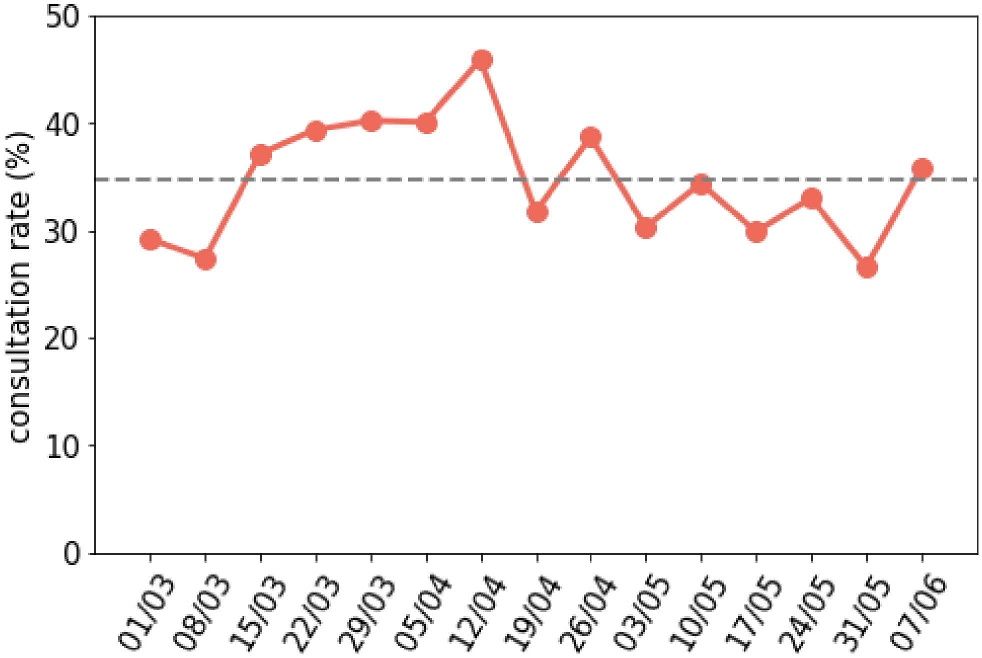

Figure S3. Consultation rate during lockdown estimated from crowdsourced data[38].

3.2. Lockdown lifted at the beginning of May

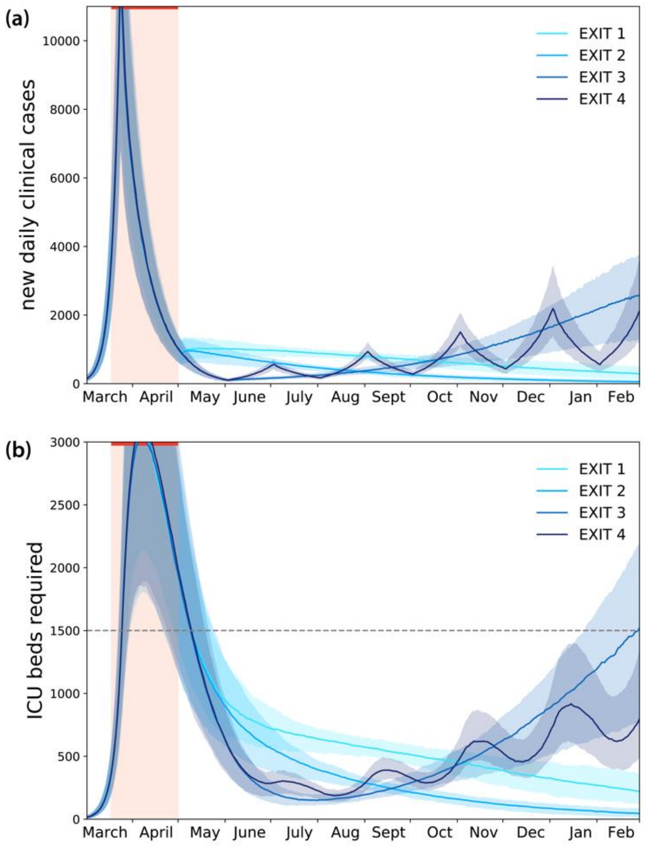

Figure S4. Simulated impact of lockdown and exit strategies with large-scale testing and case isolation, once lockdown is lifted on May 1. (a) Simulated daily new number of clinical cases assuming the progressive exit strategies illustrated in Figure 3. (b) Corresponding demand of ICU beds.

3.3. Physical contacts avoided throughout the exit strategies

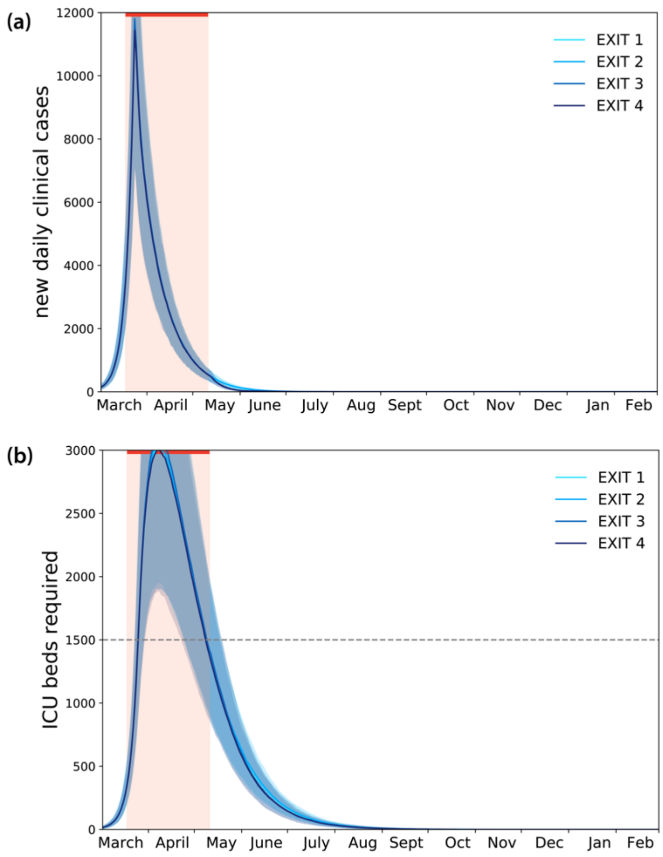

Figure S5. Simulated impact of lockdown and exit strategies with large-scale testing and case isolation, if physical contacts are avoided throughout the exit strategies. (a) Simulated daily new number of clinical cases assuming the progressive exit strategies illustrated in Figure 3. (b) Corresponding demand of ICU beds.

4. SENSITIVITY ANALYSIS

4.1. Probability of being asymptomatic 50%

Here we report the numerical results obtained assuming a higher probability of being asymptomatic ($p_a = 0.5$) compared to the main paper ($p_a = 0.2$) (Figures S6-S8).

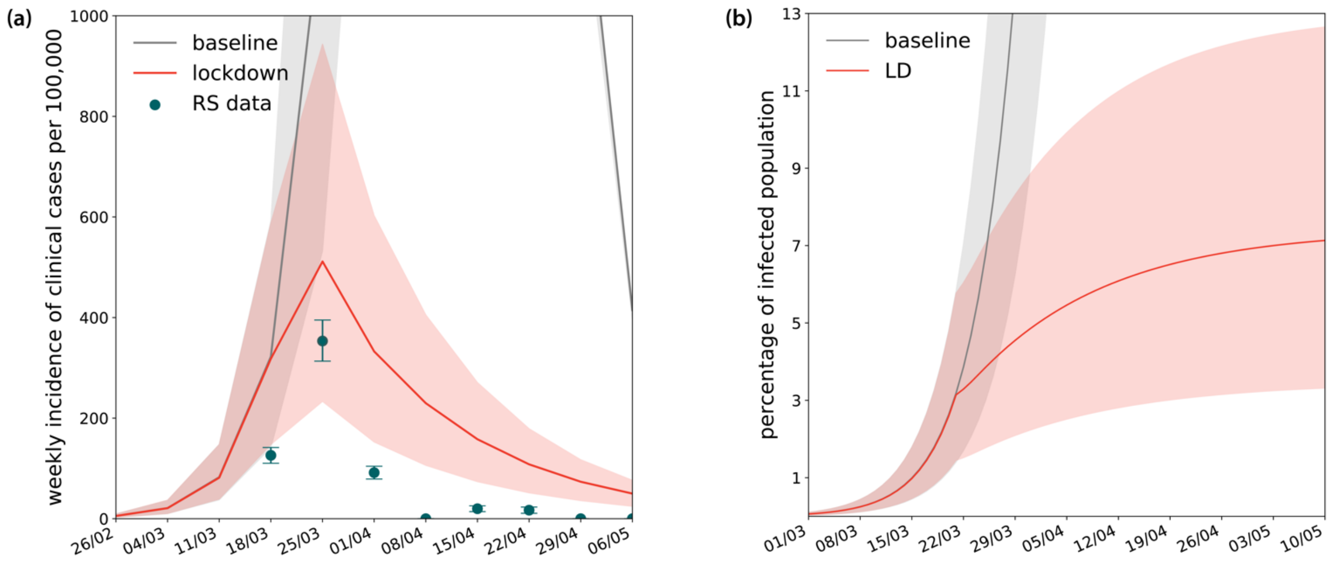

Figure S6. Estimates of weekly incidence and percentage of population infected. (a) Simulated weekly incidence of clinical cases (mild and severe) compared to estimates of COVID-19 positive cases in the region provided by syndromic and virological surveillance (Reseau Sentinelles (RS) data)[42]. (b) Simulated percentage of population infected over time. Results are shown for $p_a = 0.5$. Shaded areas correspond to 95% probability ranges.

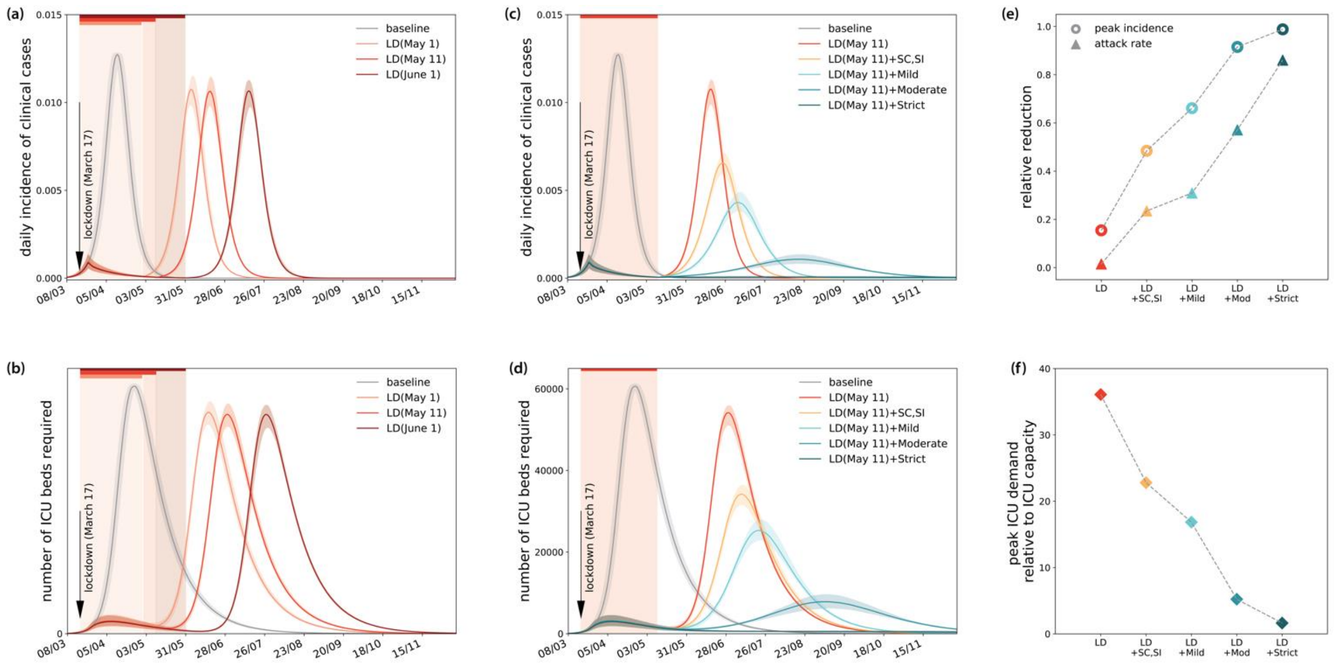

Figure S7. Simulated impact of lockdown of different durations and exit strategies. (a) Simulated daily incidence of clinical cases assuming lockdown till end of April, May 11, end of May. (b) Corresponding demand of ICU beds. (c) Simulated daily incidence of clinical cases assuming lockdown till May 11, followed by interventions of varying degree of intensity. (d) Corresponding demand of ICU beds. (e) Relative reduction of peak incidence and epidemic size after 1 year for each scenario. (f) Peak ICU demand relative to restored ICU capacity of the region (1,500 beds). In all panels, the color code is as in Table 1, and scenarios are identified as reported in Figure 3 in the main paper. Baseline scenario corresponds to no intervention. Results are shown for $p_a = 0.5$.

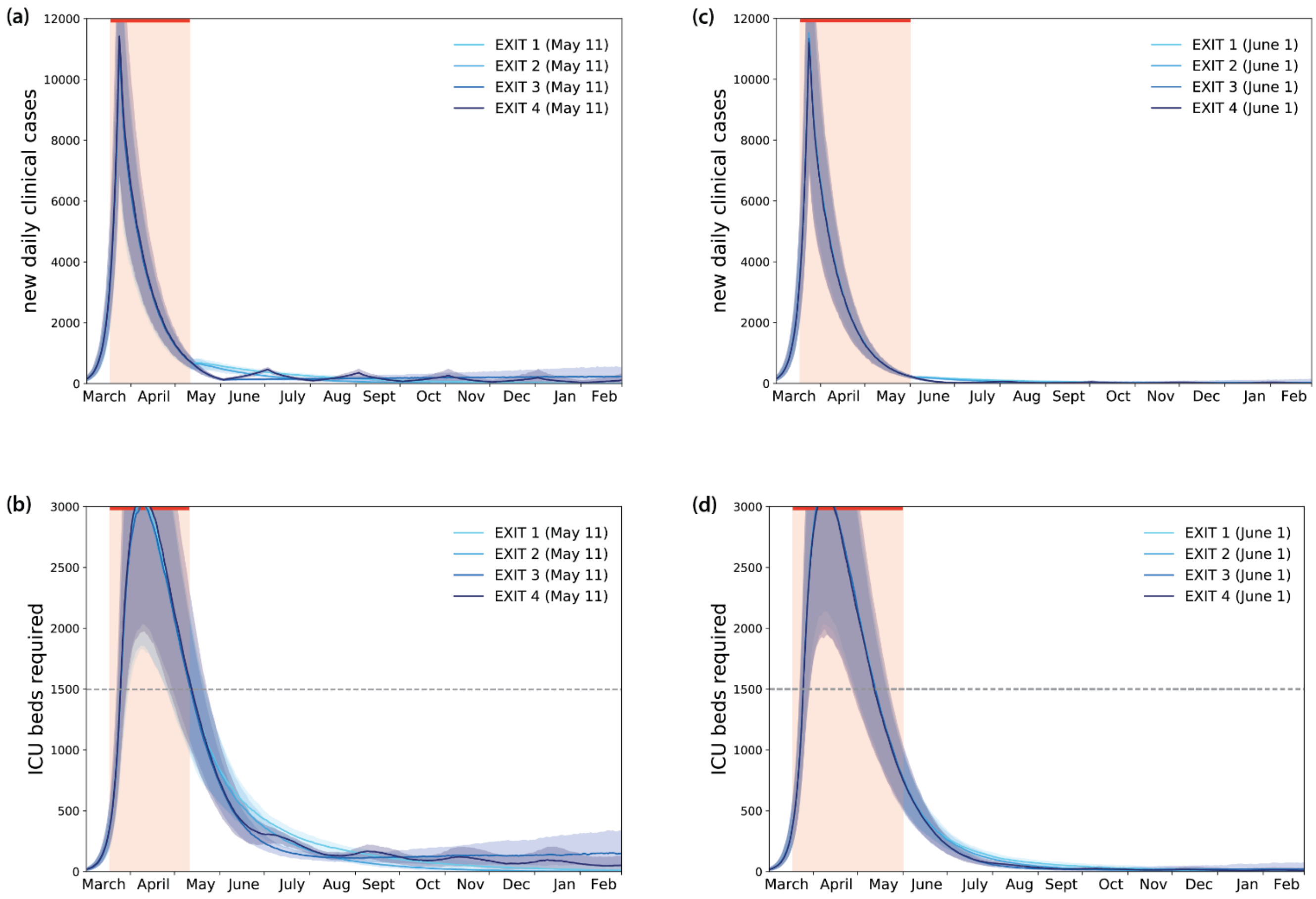

Figure S8. Simulated impact of lockdown and exit strategies with large-scale testing and case isolation. (a) Simulated daily new number of clinical cases assuming the progressive exit strategies illustrated in Figure 3. (b) Corresponding demand of ICU beds. (c) as in (a) with strategies implemented 1 month after, i.e. keeping a lockdown till the end of May. (d) Corresponding demand of ICU beds. Results are shown for $p_a = 0.5$.

4.2. Relative susceptibility of children

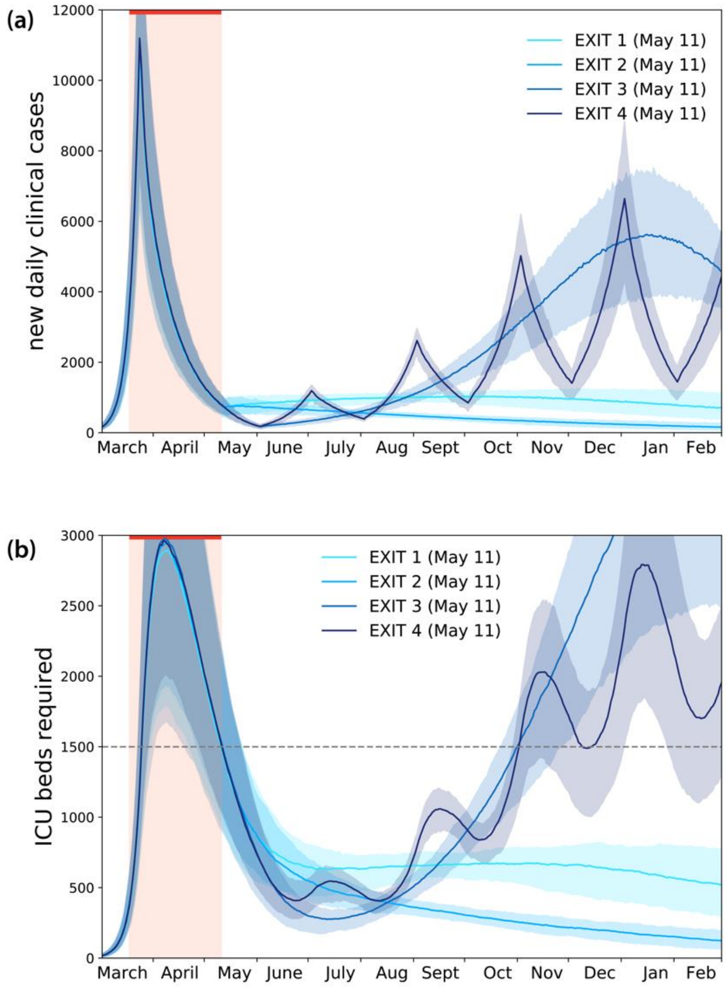

Figure S9. Simulated impact of lockdown and exit strategies with large-scale testing and case isolation, assuming that children (under 19 years of age) are 50% susceptible compared to adults. (a) Simulated daily new number of clinical cases assuming the progressive exit strategies illustrated in Figure 3. (b) Corresponding demand of ICU beds.

4.3. Relative infectivity of younger children

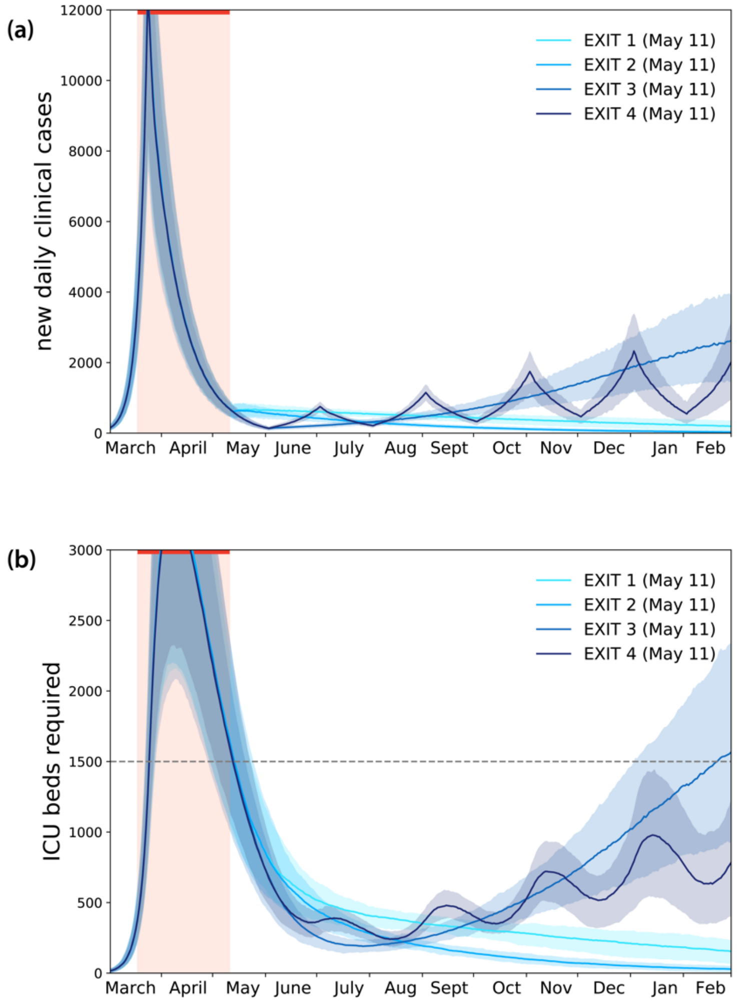

Figure S10. Simulated impact of lockdown and exit strategies with large-scale testing and case isolation, assuming that younger children (below 10 years of age) are 50% less infectious than adolescents[44]. (a) Simulated daily new number of clinical cases assuming the progressive exit strategies illustrated in Figure 3. (b) Corresponding demand of ICU beds.
